# Using data mining technology to explore homocysteine at low levels

**DOI:** 10.1097/MD.0000000000026893

**Published:** 2021-08-20

**Authors:** Fei-Ching Tseng, Tin-Chung Huang

**Affiliations:** a The Lianan Wellness Center of the Lianan Preventative Medicine Institution, Songshan District, Taipei City; b Ching-Kuo Institute of Management and Health – Graduate School of Health Industry, Zhongshan District, Keelung City, Taiwan.

**Keywords:** cancer, homocysteine, lifestyle

## Abstract

A high homocysteine level is known to be an independent risk factor for cardiovascular diseases; however, whether or not low homocysteine level contributes to any damage to the body has not been extensively studied. Furthermore, acquiring healthy subject databases from domestic studies on homocysteine is not trivial. Therefore, we aimed to investigate the causality between serum homocysteine levels and health status and lifestyle factors, particularly with a focus on low serum homocysteine levels. Additionally, we discussed a systematic methodical platform for data collection and statistical analysis, using the descriptive analysis of the chi-square test, *t* test, multivariate analysis of variance, and logistic regression.

This study was a cross-sectional analysis of 5864 subjects (i.e., clients of a health examination clinic) in Taipei, Taiwan during a general health check-up in 2017. The patients’ personal information and associated links were excluded. A sample group was selected as per the health criteria defined for this research whose data were processed using SPSS for descriptive statistical analysis using chi-square test, *t* test, multivariate analysis of variance, and logistic regression analysis.

Those working for >12 hours/day had a higher homocysteine level than those working for <12 hours/day (*P* < .001). The average serum homocysteine level was 7.9 and 8.6 mol/L for people with poor sleep quality and good sleep quality, respectively (*P* = .003). The homocysteine value of people known to have cancer was analyzed using the logistic regression analysis, revealing a Δodds value of 0.898. The percentage of subjects with a homocysteine value of ≤6.3 μmol/L, who perceived their health status as “not very good” or “very bad,” was higher than those with a higher homocysteine level. The number of subjects who perceived their health as poor was higher than expected.

The results suggest that the homocysteine level could be an effective health management indicator. We conclude that normal homocysteine level should not be ≤6.3 μmol/L. Moreover, homocysteine should not be considered as harmful and its fluctuations from the normal range could be utilized to infer a person's physical status for health management.

## 1 Introduction

Elevated homocysteine in the blood has been proven to be an independent risk factor for cardiovascular diseases.^[1–3]^ Independent risk factor, in brief, indicates that even if there is no high blood pressure, hyperlipidemia, or smoking habits, the risk of developing cardiovascular disease increases with elevated homocysteine level itself.^[4]^ Thus, researchers have coined homocysteine as the “cholesterol of the 21st century.”^[5]^ The result from searching in PubMed shows that there are >4000 scientific studies related to homocysteine from 2000 to date. Most of the existing research has focused on homocysteine from the patients’ perspective and indicated homocysteine to be a harmful substance. Moreover, there are a number of studies that have focused on the implications of a high level of homocysteine,^[6]^ but few of them have focused on the implications of a low level, and there is no consensus on the suggested range of low homocysteine levels.^[7]^ In 2004, the year seeing the peak of studies regarding homocysteine, researchers suggested that a decrease, but not an increase, in the homocysteine level may be linked to poor prognosis in patients receiving maintenance hemodialysis, which is referred to as reverse epidemiology.^[8]^ Homocysteine has weak to moderate but statistically significant correlations with certain nutritional laboratory indicators such as serum albumin, prealbumin, creatinine, and urea nitrogen. Although this may be attributed to the correlation between low homocysteine and protein energy and malnutrition^[9]^; it poses a known risk potential for poor outcome in patients receiving dialysis. Recently, certain researchers have examined hypohomocysteinemia. A notable study suggesting a high correlation between homocysteine and peripheral neuropathy has further demonstrated that 41% of patients with idiopathic peripheral neuropathy also exhibited hypohomocysteinemia.^[10]^ However, another study that examined the effects of homocysteine on inhibiting human tumor cell gelatinases, matrix metalloproteinases 2 and 9, reported that homocysteine plays a vital role in thiol groups, suggesting that the regulation of thiol levels within and outside cells is considered a potential strategy for preventing cancer.^[11]^ Based on the aforementioned results, either excessive or insufficient homocysteine can negatively impact human health. In the initial phase of the research on homocysteine, the results appear to show a similar effect as that of cholesterol; therefore, focusing on elevated homocysteine level and understanding the role it plays could be beneficial to human health management.

### 1.1 Determinants of homocysteine

**Figure 1:**
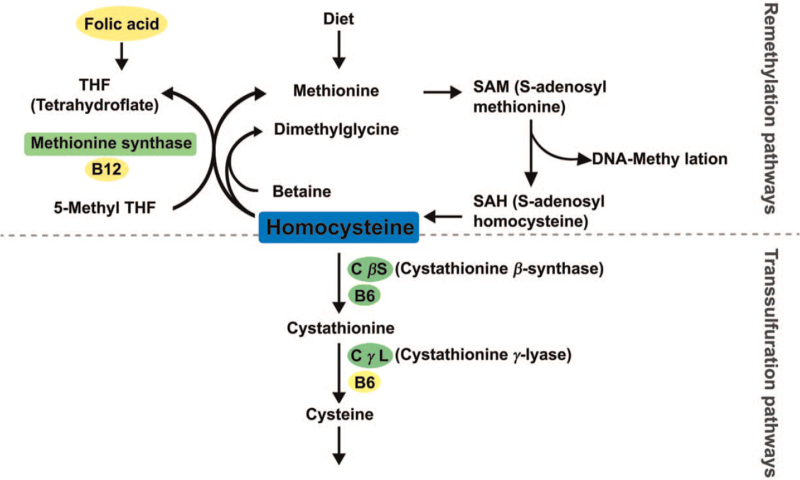
Homocysteine metabolism.

To start with, it is important to understand homocysteine based on its metabolic mechanism, which can then facilitate the analysis of its determinants. Homocysteine has 2 metabolic pathways: remethylation pathway and transsulfuration pathway,^[2]^ as shown in Figure 1 and as described below.

#### 1.1.1 Remethylation pathway

Homocysteine is the product generated from the metabolism of methionine, in which S-adenosyl methionine undergoes bonds cleavage and forms S-adenosyl homocysteine, a type of methyl donor.^[12]^ The methylation process, the bond cleavage of S-adenosyl methionine, has been proven to play a role in modulating gene expression and control.^[13]^

The homocysteine produced can use 5-methyltetrahydrofolate to provide methyl; under the catalyzation of methionine synthase and vitamin B12, methionine is again formulated from homocysteine.^[14]^ In both liver and kidney, the aid of betaine is vital in formulating methionine from homocysteine, which represents yet another intricate and complicated biochemical mechanism.^[13]^ In persons with healthy dietary intake, ∼50% of homocysteine is remethylated.^[15]^ Methionine intake directly affects homocysteine formulation and controls the ratio of transsulfuration and remethylation of homocysteine.^[12]^ In people with high protein diets, the rate of methyl loss from methionine is twice as fast as that in people with healthy diet; however, in people with low protein diets, only 10% of homocysteine undergoes transsulfuration to form cysteine.^[16]^ Other than dietary intake, methylation plays a vital role in the conversion between homocysteine and methionine.

#### 1.1.2 Transsulfuration pathway

In addition to remethylation, transsulfuration is another pathway for homocysteine metabolism. This mechanism harnesses the catalyzation of cystathionine β-synthase (CβS) with vitamin B6 and metabolizes homocysteine into cystathionine,^[14]^ it then produces cysteine from catalysis of cystathionine-γ-lyase and vitamin B6.

Homocysteine metabolism requires catalysis from numerous enzymes that need vitamin B as supplemental factors; thus, vitamin B6, B12, folic acid, and methionine^[17]^ are nutritional determinants of the homocysteine level, which is highly sensitive to the level of folic acid.^[10]^ Moreover, hereditary factors can come into play: mutation of gene C667T diminishes the ability of heat tolerance of the enzyme, 5,10-methylene-tetrahydrofolate reductase. A study has indicated that carriers of methylene-tetrahydrofolate reductase genetic mutation have significantly elevated homocysteine level.^[14]^ However, the mutation of the CβS gene can result in the deficiency of CβS, thus impacting the metabolism of homocysteine. In addition to the aforementioned metabolic mechanisms and their nutritional and genetic impacts, the determinants of homocysteine level can be associated with physiological and pathological factors and medical intervention. Age and gender are the 2 physiological factors that have been discovered, which are also factors that can be easily observed. Studies conducted outside Taiwan have demonstrated that the black population has significantly elevated homocysteine level compared to the white population.^[3]^ The difference of homocysteine level reported in different genders and women before and after menopause reveals the effect of sex steroid hormones.^[18]^ However, studying the influence of pathological factors on homocysteine levels is more challenging. According to a previous study on several diseases, such as cardiovascular diseases, dementia, depression, renal dysfunction, autoimmune diseases (rheumatoid arthritis, hyper and hypothyroidism), cancer, psoriasis,^[19]^ and intestinal diseases,^[20]^ the relation between homocysteine levels and the reduction in bone density and alcohol poisoning^[21]^ is noteworthy. However, the mechanisms of how these diseases influence the homocysteine level have not been understood. As shown in Table 1, during the process of homocysteine break down and remethylation, healthy dietary habit, vitamin B, and folic acid intake are beneficial in lowering homocysteine levels in people with hereditary enzyme defects. These factors related to the consumption of food are significant enough to influence the biochemical reactions of homocysteine. However, medication can influence homocysteine levels, which is an effect that cannot be ignored. Some essential medications are folic acid antagonists, which interfere with homocysteine metabolism. Others can disturb the normal function of vitamin B6, whereas some can cause homocysteine level to elevate by an unclear mechanism. These medications include those for sex hormone regulation, anti-epilepsy, methotrexate for cancer medication, hypolipidemic agents, blood sugar control, diuretics, and sulfonamides.^[7,19]^ In summary, homocysteine level determinants can be categorized into congenital physiological and genetic factors, and they can acquire nutritional, pathological, and medical interventions, as shown in Table 2.

**Table 1:** Homocysteine and disease.

Homocysteine	System	Disease	Correlation strength
High blood concentration	Central nervous system	Stroke	Independent risk factor
		Alzheimer's disease	Independent risk factor
		Dementia	Statistically significant correlation
		Depression	Statistically significant correlation
	Cardiovascular system	Coronary artery disease	Independent risk factor
		Myocardial infarction	Independent risk factor
		Blood clots	Statistically significant correlation
		Pre-eclampsia	Statistically significant correlation
	Gynecology system	Neural tube defect	Statistically significant correlation
	Autoimmune system	Rheumatoid arthritis with extra articular manifestation	Statistically significant correlation
	Skeletal system	Reduced bone density	Statistically significant correlation
	Gastrointestinal system	Gastrointestinal disorders	Statistically significant correlation
	Urinary system	Kidney dysfunction	Statistically significant correlation
	Cancer	Cancer	Statistically significant correlation
Low blood concentration	Peripheral nerve system	Idiopathic peripheral neuropathy	Statistically significant correlation

*Source*: Collated by this study.

**Table 2:** Determinants of homocysteine.

Factors		Item
Congenital factors	Physiological factors	Aging, male, menopause, race
	Genetic factors	Genetic alteration in metabolic enzymes (methionine synthase; MS, methyltetrahydrofolate reductase; MTHFR, cystathionine-β-synthase; CBS, and cystathionine-γ-lyase; CSE)
Acquired factors	Nutritional factors	A deficiency in cofactors (vitamin B6, B12, folate), betaine, methionine
	Pathologic factors	Decreased renal function, autoimmune system (rheumatiod arthritis, hyperthyroidism or hypothyroidism), cancer, psoriasis, gastrointestinal disorders, reduced bone density, alcoholism
	Latrogenic factors	Drugs: cholestyramine, insulin, metformin, estrogen, tamoxifen, carbamazepine, phenytoin or phenobarbital, methotrexate, thiazide diuretics, sulfasalazine

MTHFR, methylene-tetrahydrofolate reductase. *Source*: Collated by this study.

### 1.2 Homocysteine and lifestyle

Currently, there are many reasons for countries to launch propagandas in a bid to raise the awareness of healthy lifestyles. The “epidemiology analysis model,” carried out by Alan Dever in 1974, has become a predominant framework used in the process of making health policy decisions. The model proclaims that lifestyle, environment, genetics, and healthcare organization are the 4 primary factors that influence the prevalence of chronic diseases. Dever, using the data of the top 13 diseases causing the most deaths in Georgia, studied the extent of these 4 factors influencing the prevalence of chronic diseases and defined the extent in quantitative perspectives. The result shows that lifestyle accounts for 43% of the influencing weight, while heredity or human biological factors, environmental factors, and healthcare organization take up 27%, 19%, and 11%, respectively. In 1974, H.M. Lalonde, the Minister of National Health and Welfare in Canada, reported that several factors are influencing individual health, including human biology, environment, and the intervention of healthcare organizations and personal lifestyle, in which the last one can contribute to a significant improvement.^[22]^ In previous research regarding the influence of hereditary features on health, both physiological and genetic factors have been reported; in terms of the influence of the healthcare organization sector, studies have adopted different focuses, including pathological factors and medical intervention. However, in studies associated with lifestyle and environmental factors, the focal point seems to be only on nutritional factors. The possible reason for this is that most of the studies were based upon the perspective of homocysteine metabolic mechanisms.

#### 1.2.1 Dietary factor

The Hordaland homocysteine study is the first population-based study to examine dietary factors influencing homocysteine level and revealing the determinants of homocysteine in the context of lifestyle. The study shows that factors, including male, smoking, high caffeine intake, lack of exercise, and age over 65, are correlated with hyperhomocysteinemia.^[23]^ According to the cohort study conducted by Framingnam, alcohol intake, and creatinine level are 2 other determinants of high homocysteine level.^[24]^ Moreover, there is research suggesting that, in addition to the main lifestyle determinants affecting homocysteine level such as smoking, vitamin supplement, and caffeine consumption, the time and circumstance of the last meal of a day should be factored into consideration.^[25]^ From the viewpoint of homocysteine metabolic mechanisms and its determinants, vitamin B12 deficiency can cause homocysteine level to elevate, indicating that vegetarians have a higher predilection to have vitamin B12 deficiency than non-vegetarians.^[26]^ A study, done by systematic review and meta-analysis, analyzed 11 cross-sectional studies and 17 studies between 1999 and 2010, in which 3230 subjects were included, and compared the difference of homocysteine and vitamin B12 level between vegetarian and non-vegetarian populations. Only 2 of the abovementioned studies reported no significant differences in homocysteine and vitamin B12 levels in blood between vegetarians and non-vegetarians.^[27]^ An enzyme requires a certain amount of water to maintain its natural structure such that its full function can be exhibited. Water can be a substrate or a product of enzymatic reactions that affects enzyme conversions in different ways. It is known that regardless of the types of reactions, enzymes will function to the fullest extent if the optimal level of water is contained.^[28]^ The metabolic mechanisms of homocysteine involve several enzymes; however, very few studies have investigated whether water intake habits and daily water intake volume can be determinants in homocysteine level.

#### 1.2.2 Activity factor

Homocysteine level is an independent risk factor for cardiovascular diseases. The 7 risk factors for cardiovascular disease, defined by the American Heart Association, are smoking status, blood pressure, cholesterol level, body mass index (BMI), blood glucose level, physical activity, and diet.^[29]^ In terms of lifestyle, the key factors for cardiovascular diseases are smoking, physical activity, and a healthy diet. Physical activity refers to any form of physical exercise that can train muscle or demand more energy than resting such as walking, running, dancing, swimming, yoga, or gardening.^[30]^ In a quasi-experimental study, observations were made on the effects of exercise in sedentary men and their visceral fat, homocysteine, C-reactive protein, and blood lipid levels. The exercise program was implemented for 8 weeks, when the subjects were asked to perform aerobic exercise for 60 minutes with the target heart rate set at a maximum heart rate of 75% to 85% for 3 times per week. The result revealed a significant reduction in visfatin and lower homocysteine and C-reactive protein levels, although not statistically significant (*P* > .05).^[31]^ Another study explored the change of homocysteine level in primary dysmenorrhea patients after yoga exercise, but no significant findings were identified.^[32]^ The findings of these studies seem to indicate that physical exercise does not influence homocysteine concentration in blood. The mental and physical exertion during work requires more energy than that during rest and thus has an impact on one's health. Therefore, work should be considered as a type of activity factor. In the review of general health and physical examination records, the data of work hour count is a critical component because of its high relevance to the health of workers. A systematic review investigated the relationship between health and long working hours. In 17 published articles and 19 studies between 1995 and 2012, having long working hours is associated with depression, anxiety, the reduction of sleep quality, and the incidence of coronary heart diseases.^[33]^ Currently, homocysteine levels are definitely an independent risk factor for cardiovascular diseases,^[34]^ and the work hour count is related to coronary heart diseases; however, there is no study in the country focusing on the relationship between work hour count and homocysteine level. This study aims to investigate what role do activity factors play in a healthy lifestyle, as well as to determine what influence does the number of average daily working hour has on homocysteine levels.

#### 1.2.3 Sleep factor

In the last few years, studies have revealed how sleep affects the progression of Alzheimer's disease, and the fact that high level of homocysteine plus sleep deprivation can lead to higher oxidative damage.^[35]^ The quantity of sleep and the problems during sleeping, such as obstructive sleep apnea (OSA), can impact the quality of sleep. Intermittent hypoxia caused by OSA may induce severe hypoxemia, which may be closely associated with coronary heart diseases and prompt researchers to study the relationship between OSA and homocysteine. Analysis has revealed that patients have significantly higher homocysteine level in blood compared to that in the healthy control group. The phenomenon of higher homocysteine level is even more evident in patients with moderate or highly severe OSA symptoms.^[36]^ In 2014, Beydoun et al collected data on nutritional biomarkers and used comprehensive selection tools to determine several indexes representing sleep quality, expanding the sleep-related research from the previous main focus of sleep quantity or sleep disorders.^[37]^ In addition to sleep quantity and sleep disorders, disorders of sleepiness were highlighted. The result shows only sleep quantity has a significant effect on homocysteine level (*P* = .011), where the subjects sleeping for <5 hours exhibited significantly higher homocysteine level than the other groups.^[37]^ However, sleep quality is a term extensively used by researchers, clinical doctors, and the general population, yet there is no consensus on the definition of this term.^[38]^ There are very few studies related to homocysteine level and sleep quality. Preventative medicine emphasizes on the correction and maintenance of lifestyle; moreover, it is a common mode of intervention in health-related studies. In reality, there is no single causality between chronic diseases and lifestyle. The effects of a healthy lifestyle and its impact on homocysteine level in blood are a complex issue that requires further investigation.

### 1.3 Purpose

Based on the background and motivation listed above, the purpose of this study included:

1. to explore the correlation between physiological factors and homocysteine values,
2. to explore the relationships between lifestyle habits and homocysteine values,
3. to explore the correlation between self-perceived health status and low homocysteine values,
4. to explore the relationship between cancer patients and hypohomocysteinemia, and
5. to provide research results as a reference for the health management classification model.

## 2 Methods

The main aim of this study was to determine the homocysteine levels of subjects without cardiovascular and cerebrovascular diseases. Most clients of the health check facilities are healthy or subhealthy people. Therefore, we used purposive sampling of clients of the health examination clinic in Taipei City, Taiwan in 2017.

This study is a cross-sectional analysis with 5864 subjects who were clients of a health examination clinic in Taipei, Taiwan during a comprehensive health check between January 1, 2017 and December 31, 2017. The personal information and links on their questionnaire were retrieved; subsequently, the health check reports with their “Healthy Status Questionnaire” were analyzed to explore the relationship between serum homocysteine levels and the data from the questionnaires. This study was reviewed and approved by the Human Research Ethics Committee of Fu Jen Catholic University (approval number: C107183). Informed consent was waived by the by the Fu Jen Catholic University Institutional Ethics Committee.

The Healthy Status Questionnaire contained a behavior-description survey, which enables the health examiners to screen and identify clients’ health risk and then suggest and discuss the health check requirement during the health check process. After the health check, a health intervention plan is built. This ISO 9001:2015-certified clinic has rigorous specifications for the health check process. Questionnaires were administered to a health examinee by a nurse on the health examination day. The process of the questionnaire was as follows: the questionnaire was initially attempted by the examinee. If any question was unanswered, a nurse would interview the examinee during the health examination day to complete the questionnaire. Withholding or refusing to answer any medical information was acceptable, and the answer was considered as empty or “refused to answer.” According to the requirements of this study, we analyzed 3 sections of the questionnaire: lifestyle, self-perceived health status, and medical history. The “eating-habits factors” in the “lifestyle” section includes the habit of drinking coffee, alcohol consumption, and vegetarianism. The “activity factors” include working hours and exercise habit. The “sleep factors” include the length of sleep time and the quality of sleep. The factors of “self-perceived health status” include personal health status and self-perceived symptoms. The factor of “medical history” evaluated whether the examinee was diagnosed with cancer. The exclusion factor in this study was the diagnosis of cardiovascular disease.

An effective group was thereby selected, which included clients free from myocardial infarction, coronary artery diseases, arteriosclerotic heart diseases, stroke, and Alzheimer's disease, according to the “medical history” from the questionnaire. The results were obtained from evaluating 5668 clients.

**Figure 2:**
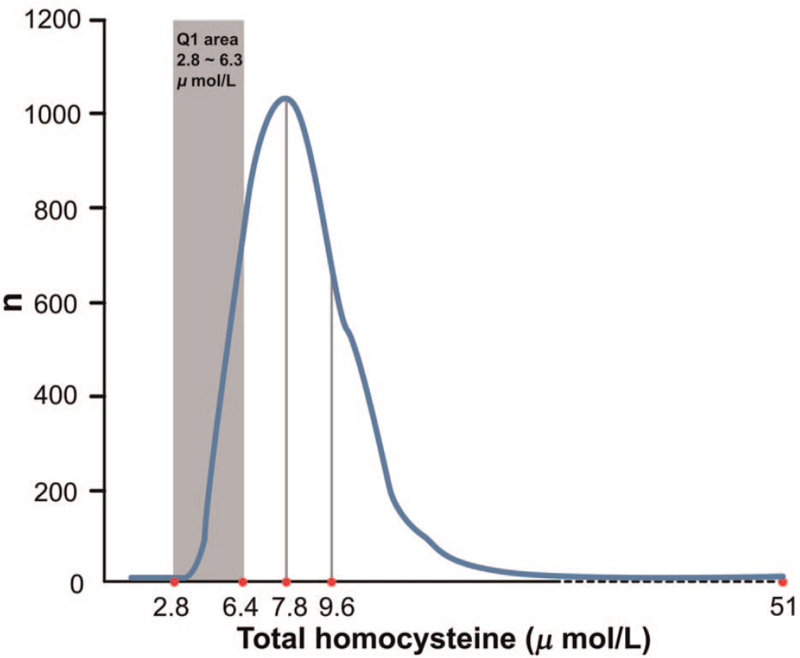
Distribution of total serum homocysteine and quartiles of homocysteine in 5668 samples. Cases with total serum homocysteine of >50 μmol/L are represented by 51 (n = 4).

The homocysteine samples of this study were retrieved from the subjects who were fasting for >8 hours daily. The samples were then analyzed using the Architect i2000 system. The analysis method of homocysteine was chemiluminescent microparticle immunoassay (CMIA). Using CMIA, the homocysteine upper level is 50 μmol/L; thus, there were 4 subjects with their homocysteine value of ≤50 μmol/L, which was redefined as 51 μmol/L. To observe the population with a low serum homocysteine value, it is necessary to group the samples into quartiles, as shown in Figure 2. The statistic differences from Q1 to the other 3 groups can then be analyzed.

### 2.1 Sample size calculation

For sample size calculation, the following were used as reference: the sample sizes of

(1) a longitudinal systematic review by Kuo et al in 2005 and
(2) a systematic review and meta-analysis of 17 cross-sectional studies by Obersby et al in 2013.

In addition, the data used in this study came from Taipei City, Taiwan. If the homocysteine level of Taipei City's healthy population was used as the standard, the official website's calculator was used to estimate Taipei City's healthy sample population as the sample size. These 3 points were used in combination for sample size reference.

The 2005 longitudinal systematic review was a systematic review on the association between homocysteine level and age. For data collection, the authors searched the Medline database (January 1966–March 2004) and included those above 55 years of age as the study subjects. Besides, the researchers employed a standardized format to examine the data in the related articles (Kuo et al, 2005) to collect 16 longitudinal studies that used the homocysteine level as a cardiovascular disease risk factor. The included studies had sample sizes in the range of 104 to 4766 subjects. The other article was a systematic review and meta-analysis. The investigators collected 17 cross-sectional studies between 1999 and 2010 that compared the plasma total homocysteine levels of vegetarians and non-vegetarians. They collected 3230 study subjects (Obersby et al, 2013). We hope that the results of this study can be used as an index to estimate and evaluate the health status of Taipei's population. We availed the online services of Creative Research Systems and used the calculator provided by the website. The following settings were used: 95% confidence level, sampling error within 3 decimal points, population as Taipei City's average population between January and December, 2017 at 2,688,430 (Department of Civil Affairs, Taipei City Government, 2019). The estimation suggested that Taipei City's healthy sample size should be at least 1067 subjects.

### 2.2 Statistical analysis

The data was processed in the SPSS software for statistics analysis. We used analysis of variance and Scheffe's method of multiple comparisons to analyze factors such as, eating habits, activity, and sleep. We distributed the data of self-perceived health status into 4 groups for further analysis. Further, logistic regression analysis was used to analyze the homocysteine levels of those with or without cancer.

## 3 Results

This study is different from other studies in that the data of sick people used were collected by the National Health Insurance Database. To obtain the data pertaining to healthy group (including subhealthy group), data mining was performed on the homocysteine data based on the original source from a health examination center in Taipei City in 2017. In the remaining 5668 homocysteine sample data, quartile grouping was performed, and the obtained average values of each grouping were Q1 = 5.4, Q2 = 7.1, Q3 = 8.5, and Q4 = 11.8 (Table 3). Subsequently, these quartile data were used in conjunction with those in another study with the same laboratory method held by the Chin-Shan Community Cardiovascular Cohort of National Taiwan University in which subjects were people having diastolic dysfunction. The quartile value they obtained (Q1 = 7.6, Q2 = 9.3, Q3 = 11.2, and Q4 = 14.75) was significantly higher than that obtained in this study. This comparison procedure suggests that researchers should not compare the homocysteine level distribution of healthy people to nonhealthy people. As shown in Table 3, considering that the source of data of this study was from a health examination center, it could be assumed that the results could reflect the serum homocysteine distribution of a healthy group (including subhealthy group). Furthermore, the correlation between homocysteine values with subjects’ physiological factors, lifestyles, self-perceived health status, and medical history of cancer was observed and analyzed.

**Table 3:** Quartile groups of homocysteine.

Homocysteine	Q1 n = 1371 μmol/L	Q2 n = 1417 μmol/L	Q3 n = 1407 μmol/L	Q4 n = 1473 μmol/L
Mean (SD)	5.4 (0.7)	7.1 (0.4)	8.5 (0.5)	11.8 (3.6)
Min–Max	2.8–6.3	6.4–7.7	7.8–9.4	9.5–51

### 3.1 Distribution of homocysteine

**Figure 3:**
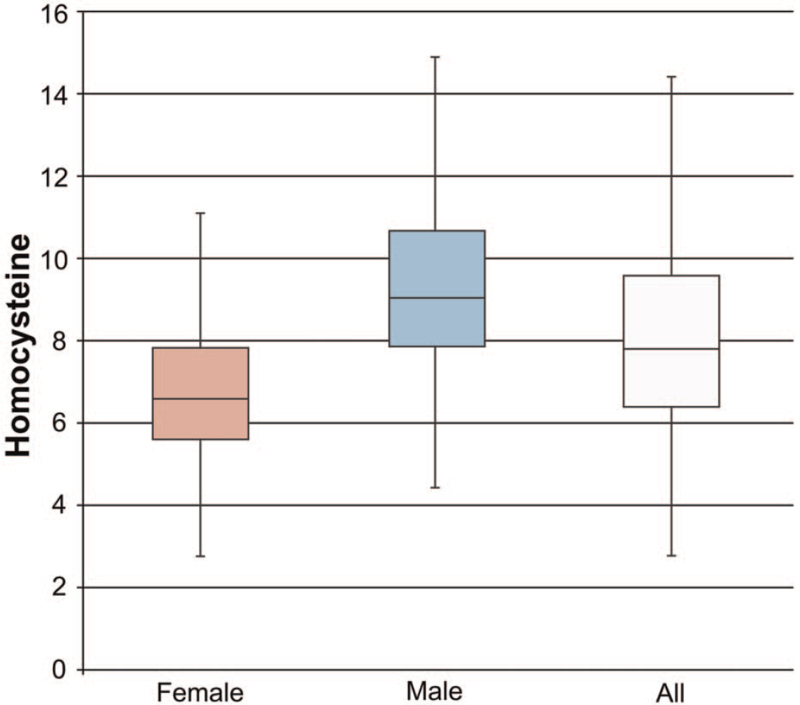
Boxplot of the homocysteine levels.

For the 5668 samples, the homocysteine value was in a positively skewed distribution (skewness = 4.246, skewness standard error = 0.033) with an average value of 8.2 μmol/L and a median of 7.8 μmol/L. Note that the mode is 6.9 μmol/L, the standard deviation is 3.0 μmol/L, the minimum is 2.8 μmol/L, and the maximum is 51.0 μmol/L (Fig. 3).

### 3.2 Exploratory data analysis for physiological factors

#### 3.2.1 Gender and age

In this study, the interaction between gender and age groups was observed. The resulting gender and age distributions were different from the past studies conducted in Europe and the USA. Moreover, there were no significant differences between the homocysteine level and age group for the male group; however, the difference was obvious for the female group (Table 4).

**Table 4:** Gender, age, and average values of homocysteine.

Gender	Age group yrs	Code	n	tHcy (μmol/L)		*P*	Sheffe Code
				Means	(SD)		
Men	<30	1	210	9.7	(4.1)	.06	N/S
	31–40	2	539	9.9	(4.3)		
	41–50	3	749	9.7	(2.8)		
	51–60	4	788	9.4	(2.8)		
	61–70	5	398	9.8	(3.5)		
	>71	6	68	10.0	(2.7)		
	All age group		2752	9.7	(3.3)		
Women	<30	1	292	7.0	(2.0)	.00	6 > 5 > 2.3
	31–40	2	697	6.7	(1.9)		
	41–50	3	776	6.6	(1.7)		
	51–60	4	731	7.0	(2.0)		
	61–70	5	366	7.5	(2.1)		
	>71	6	54	8.7	(2.6)		
	All age group		2916	6.9	(1.9)		

N/S indicates that there is no significant difference in multiple comparisons after the Sheffe method. ^∗^*P* < 0.05, ^∗∗^*P* < 0.005, ^∗∗∗^*P* < .001.

#### 3.2.2 Body mass index

Although homocysteine is an indirect indicator of protein energy malnutrition, it could lead to a misunderstanding that people with underweight tend to have lower homocysteine levels. The BMI classification of this study was defined by the Health Promotion Administration, Ministry of Health and Welfare. BMI was stratified and tested using Scheffe's method as per the 4 groups: underweight, normal, overweight, and obese. According to the statistical values, there was a significant difference between the serum homocysteine value for those with BMI ≥ 24 kg/m^2^ (obese or overweight) and those with BMI ≤ 24 kg/m^2^ (normal or underweight).

### 3.3 Exploratory data analysis for eating-habits factors

The correlation between habits of coffee drinking, water drinking, alcohol consumption, vegetarianism, and homocysteine level is to be detected using multivariate analysis of variance. Before the subjects of cardiovascular diseases were excluded, there was no significant difference between the habits listed above and homocysteine levels. Because homocysteine has been proven as one of the risk factors to cardiovascular diseases, the subjects diagnosed with cardiovascular diseases were excluded from the effective group. The statistic significant differences were observed after the effective group data were analyzed. The significant differences in serum homocysteine levels were observed in different alcohol drinking group, and also in the group that was or was not vegetarian. The result demonstrated subjects who consumed alcohol almost every day had an average homocysteine level of 8.9 μmol/L in their blood. It was significantly greater than that of the group of nonalcoholics, that is, 7.9 μmol/L (*P* < .001). The vegetarians had their homocysteine value of 8.6 μmol/L, which was significantly ≥8.1 μmol/L in the non-vegetarian group (*P* = .025). Moreover, the result demonstrated that coffee drinking habit and the water drinking habit were the interference factors of alcohol consumption.

### 3.4 Exploratory data analysis for activity factors

To examine the factors of exercise habit and working hours, this study demonstrated that only working hours had a significant difference in serum homocysteine values. In fact, the group working < 10 hours a day had lower average homocysteine levels than the group working for >12 hours per day. Therefore, employers and medical professionals are recommended to pay attention to both the hazards of overwork and long-term workload (Table 5). According to the study results, the recommended working hour should be <12 hours a day. Many researchers conducted in-depth studies on the correlation between exercise and homocysteine values. One such similar study is the Alavizadeh et al study that observed the effectiveness of exercise intervention on several cardiovascular risk factors and demonstrated no statistically significant on the change in homocysteine levels.^[31]^ Our study came up with the same result that there was no statistically significant difference between different exercise habit groups and serum homocysteine levels. Thus, this study confirmed that exercise is not the impact factor of homocysteine.

**Table 5:** Working time and homocysteine.

	n	Scheffe (collection of Alpha = 0.05)	
Working time/day h		1	2
		Means (SD) (μmol/L)	
<8	1009	8.1 (2.9)	
8–10	1853	8.3 (2.9)	
10–12	523	8.7 (3.8)	8.7 (3.8)
>12	178		8.9 (3.9)

Two-way ANOVA, *F* = 4.999, *P* = .00.

### 3.5 Exploratory data analysis for sleep factors

In the context of sleep factors, the results showed that the factor that causes significant differences was not the length of sleep time but the quality of sleep in homocysteine values. Moreover, there was no interaction between the length of sleep time and the sleep quality. Furthermore, it was reported that when the sleep quality is poor, the average homocysteine concentration in the blood is lower than that with good sleep quality (Table 6).

**Table 6:** Sleep quality and homocysteine.

		Scheffe (collection of Alpha = 0.05)		
Sleep quality	N	1	2	3
		Means (SD) (μmol/L)		
Poor	1200	7.9 (2.7)		
Ordinary	1679		8.3 (2.7)	
Good	783			8.6 (3.6)

Two-way ANOVA, *F* = 7.232, *P* = .00.

### 3.6 Self-perceived health status

From previous studies, the low homocysteine level group was usually overlooked. As for the expected value, the results showed that the Q1 group and the Q4 group had the same result, while the Q2 group and the Q3 group had poorer health and lower expected value (Table 7). In terms of the number of symptoms, the Q1 group (tHy = 2.8–6.3 μmol/L) had significantly more symptoms than the Q3 and Q4 groups. Moreover, it is confirmed that the Q4 group is not necessarily the only high-risk group. These results suggest that future studies should observe the effects of low homocysteine to the body.

**Table 7:** Self-perceived health status and homocysteine Chi-square test.

Homocysteine (M ± SD) μmol/L		Quartiles of homocysteine					
		Q1 n = 1371 5.4 ± 0.7	Q2 n = 1417 7.1 ± 0.4	Q3 n = 1407 8.5 ± 0.5	Q4 n = 1473 11.8 ± 3.7	Total	Chi-square value
Very satisfied	n (%)	195 (21.9)	251 (28.2)	225 (25.3)	219 (24.6)	890	14.446^∗^
Satisfaction	Expected value	224.8	226.2	218.9	220.1		
Fair	n	687 (25.6)	669 (25)	673 (25.1)	650 (24.3)	2679	
	Expected value	676.5	680.9	658.9	662.7		
Not good	n	191 (28.1)	160 (23.5)	147 (21.6)	182 (26.8)	680	
Very bad	Expected value	171.7	172.8	167.2	168.2		

Chi-square test, *P* = 0.025, Cramer's *V* = 0.041.

∗ *P* < 0.05.

### 3.7 Cancer patients

Note that an independent sample *t* test was used to observe the difference in serum homocysteine concentration between the 2 groups: those diagnosed with and without cancer. The average serum homocysteine value of subjects diagnosed with cancer was 7.6 μmol/L, which was ≤8.3 μmol/L, that is, the average value of subjects diagnosed without cancer. Subsequently, the chi-square test was applied between the subjects who had cancer and the quartile group of fasting.

Serum homocysteine of subjects: the results of having cancer of Q1 group (tHy = 2.8–6.3 μmol/L) and Q2 (tHy = 6.4–7.7 μmol/L) were higher than their expected values (Table 8).

**Table 8:** Cancer patients in quartiles groups of homocysteine.

	Quartiles of homocysteine					
(μmol/L)	Q1 2.8–6.3 n (%)	Q2 6.4–7.7 n (%)	Q3 7.8–9.4 n (%)	Q4 9.5–51.0 n (%)	Total n (%)	Chi-square value
No cancer	1315 (23.9%)	1368 (24.9%)	1371 (24.9)	1447 (26.3%)	5501 (100%)	15.436^∗^
Expected	1330.6	1375.3	1365.5	1429.6		
Cancer patients	56 (33.5%)	49 (29.3%)	36 (21.6%)	26 (15.6%)	167 (100%)	
Expected	40.4	41.8	41.5	43.4		

∗ *P* < .005.

The logistic regression analysis showed that Δodds = 0.898, which is <1. Moreover, when the value of homocysteine increased by 1, the odds value of cancer was increased by 0.898 times.

## 4 Discussion

Homocysteine level in blood can be considered as an indirect nutritional index. Moreover, it can be related to alcohol intake, vegetarian diet, work hour count, sleep quality, personal health status, numbers of self-perceived symptoms, and cancer-related risks. Considering the dietary factor that reflects a lifestyle, the study results suggest that those who consume alcohol almost daily have higher homocysteine levels than the nondrinking population. Vegetarians had higher homocysteine levels in blood than non-vegetarians. As for the activity factor, those who worked for ≥12 hours per day has higher average homocysteine concentration than those who worked for <10 hours per day. Moreover, persons with poor sleep quality had an average blood homocysteine concentration of 7.9 μmol/L, which is ≤8.6 μmol/L (*P* = .003) with good sleep quality. From a self-perceived health perspective, many studies associate elevated homocysteine level with diseases. Data from surveys on self-health assessment showed that in homocysteine quartiles, those who indicated their self-health as “Not Good” to “Bad,” Q1 group (lower quartile) and Q4 group (higher quartile) had statistics higher than the expected value. As per the number of self-perceived symptoms, Q1 group had a significantly higher self-perceived symptom count than other groups (Sheffe test, *P* < .001). From the viewpoint of disease history, the result of logistic regression reveals the odds of persons who proclaim self-indicated cancer against homocysteine level, Δodds = 0.898 < 1. As per the above analytical results, the formation and metabolism of homocysteine are associated with the balancing mechanisms of the body, such as cholesterol and glucose, the excess amount of which poses a burden to health, whereas deficit induces metabolic problems. These data indirectly state that blood homocysteine level should be one of the indexes of health management. Homocysteine should not be considered as a purely harmful substance; instead, the conditions of either excess or deficit of it should be considered equally important. For those with blood homocysteine concentration of ≤6.3 μmol/L, their self-perceived symptoms are more than those with higher homocysteine level. The occurrence of self-perceived poor health in those with low homocysteine level is higher than the expected value. In this study, the ideal blood homocysteine concentration lower range is determined to be 6.3 μmol/L. For those with blood homocysteine level of ≤6.3 μmol/L, the analysis should be performed on their homocysteine metabolic mechanisms and determinants such that additional evaluation of appropriate usage of protein intake and methionine supplement can be performed. For those with elevated homocysteine level, by examining nutritional, genetic, physiological, pathological, and medical intervention factors, conditions such as folic acid and vitamin B deficiency because of dietary habits or drugs can be assessed for nutritional supplements.

## 5 Limitations

This research is a cross-sectional study that aims to quickly understand the blood homocysteine signatures, distribution, and lifestyle habits as well as their correlations among the population of Taipei City in 2017. However, this study only analyzed data from 2017 and lacked a long-term database; thus, the differences in generations and trends are difficult to assess. The health survey used by the health examination clinic of the preventative medical facility was designed for health-related habits and status survey, and not for academic research, because its purpose is to help the medical staff of the health examination clinic to quickly evaluate the subjects’ health risks and the suitability of the health check procedures to the subjects. The purpose of this research is to use data analysis techniques for the initial probing and to study the relationships between the current health information and homocysteine. Moreover, the arrangement of experimental variables presented a challenge to exclude all factors that may completely affect homocysteine expression. The health examination facility that provided the data used in this study uses CMIA to test for homocysteine. The results obtained by this test approach show high relevance to the results from high performance liquid chromatography; however, the data of concentration is on an average 5% to 10% lower than that for high performance liquid chromatography tests. Such a test method has acceptable precision (CV = 6%–8%) and has been considered an acceptable choice for conventional clinical laboratories.^[39]^ Unfortunately, there is no standardized approach either in Taiwan or in the international community to conduct studies on homocysteine concentration. There are still testing laboratories in Taiwan that use different formats and different platforms from other countries,^[40,41]^ rendering analytical results difficult to be objectively benchmarked. The results of this study can provide reference models regarding the use of the CMIA method in research, which is particularly suitable for fundamental investigations on a large scale of population.

## Acknowledgments

I would like to express my gratitude to people at Lianan Wellness Center who gave the golden opportunity to reach this wonderful environment with such abundant data to complete this study. First, I would like to thank Dr Nai-Yuan Cheng, the superintendent of Lianan Wellness Center, who gave the permission for this study and provided several intellectual discussions about this research. A special note of thanks to Po-Lin Chen in the Department of Information of Lianan Wellness Center for assistance in identifying the proper data for this study and helping to remove the personal information and link. Finally, I am grateful to all of those with whom I have had the pleasure to work during this study.

## Author contributions

**Formal analysis:** Fei-Ching Tseng.

**Investigation:** Fei-Ching Tseng.

**Methodology:** Fei-Ching Tseng.

**Supervision:** Tin-Chung Huang.

**Writing – original draft:** Fei-Ching Tseng.
